# Comparative study of vulva and abdominal skin microbiota of healthy females with high and average BMI

**DOI:** 10.1186/s12866-019-1391-0

**Published:** 2019-01-17

**Authors:** Rebecca Vongsa, Doug Hoffman, Kristin Shepard, David Koenig

**Affiliations:** Kimberly Clark Corporation, 2100 County Rd II, Neenah, WI 54956 USA

**Keywords:** Body mass index, pH, Microbiome, Microbiota, Vulva, Abdomen, And *Lactobacillus*

## Abstract

**Background:**

Obesity is known to modulate human health in a number of ways including altering the microbiome of the gut. Very few studies have examined the how obesity may affect the microbiomes of sites distant to the gut. We hypothesized that vulva and abdominal skin may be especially susceptible to body mass index (BMI)-induced alterations in biophysical properties and the microbiome due increased maceration and skin folds at those sites. The aim of this study was to determine if high BMI (≥30) was associated with alterations in the biophysical properties and microbiomes of vulva and abdominal skin.

**Results:**

The vulvar microbial communities of healthy reproductive-aged females were examined using 16S rRNA sequencing techniques. Our results show that vulvar pH of women with high body mass index (BMI) was statistically higher than that of women with average BMI. Phylogenetic analysis of the vulvar microbiota indicated that women with average BMI have a predominately *Lactobacillus*-dominated flora, whereas women with high BMI and higher pH were predominately colonized by *Finegoldia* and *Corynebacterium*. This BMI-associated shift in microbiota was not observed in samples collected from the exposed skin around the belly, indicating the effect is not global.

**Conclusion:**

These results indicate that physiological changes associated with changes in BMI may modulate the vulva microbiome.

**Electronic supplementary material:**

The online version of this article (10.1186/s12866-019-1391-0) contains supplementary material, which is available to authorized users.

## Background

The human body is inhabited by communities of microbes that have adapted to particular anatomical sites [1]. The microbial populations found in the gut and at sites outside of the gut like the skin and vagina have been linked to a variety of human health conditions including inflammatory bowel disease, ulcers, colorectal carcinoma, vaginosis, depression, psoriasis, and obesity [2–6]. With the prevalence of obesity on the rise in many countries, an increasing number of studies have been done to elucidate the mechanism and consequences of this condition. Several studies revealed a strong link between obesity and the composition of the gut microbiome [7–10] and one study demonstrated a link between obesity and the microbiome of the internal female urogenital tract [11]; however less known about the effect of obesity on the microbiomes of other anatomical locations.

The skin microbiome has been well-characterized and is known to harbor distinct site-specific microbial communities that are influenced by the biophysical properties of the niche like moisture and pH [12–17]. The vulva is a unique region of skin that serves as a transition between the cutaneous epithelium of the skin and the mucosa of the female urogenital tract. To date, relatively few studies have been performed to characterize the microbes that colonize the vulva of healthy females. Vulvar microbial communities are thought to be of clinical importance because they regulate the proliferation of non-indigenous flora, including pathogens that can cause infection and may also affect the comfort of the urogenital area. Therefore, factors that modulate the vulvar microflora may affect comfort and health of the vulvar area.

Obesity has been shown to affect female physiology by increasing plasma estrogen levels, elevating systemic inflammation, and reducing immune function [18–20]. We hypothesized BMI-induced physiological changes would result in altered biophysical properties of the skin which, in turn, would modulate the microbiome at those site. This study focused on abdominal and vulvar skin because it was hypothesized that these sites may be especially susceptible to BMI-induced changes in biophysical properties and microbiota structure due to increased skin folds, occlusion, and maceration. Therefore, it was the aim of this study to determine whether obesity, defined as a BMI of 30 or greater, was associated with alterations in the biophysical properties and microbiome of the vulva and abdominal skin.

## Results

### BMI status was associated with higher pH in the labium

In order to determine whether BMI altered the biophysical properties of the skin and vulvar region, the pH and trans-epithelial water loss (TEWL) of outer labia and exposed abdominal skin were measured. Results in Table 1 show that women in the high BMI group had a statistically higher pH in the vulvar region than women in the average BMI group. These results are consistent with those from a previous study that reported a positive correlation between vaginal pH and BMI [21]. The pH of the exposed abdominal skin was not different between the low and high BMI subjects. Table 2 shows that TEWL of exposed skin was statistically lower in the high BMI group, although still within ranges that are generally regarded as healthy [22, 23]. These data indicate that BMI may alter specific biophysical properties of cutaneous epithelium in a site-specific manner.

**Table 1:** pH of exposed abdominal skin and outer labia in high BMI and normal BMI subjects

Site	BMI status	mean	st. dev.	*p* value
exposed skin	average BMI	6.069	1.034	0.080
	high BMI	6.589	0.965	
outer labia	average BMI	5.925	0.648	0.035
	high BMI	6.357	0.582	

*p*-values reported in Table 1 were calculated using a Student’s t-test (95% confidence interval)

**Table 2:** TEWL of exposed abdominal skin and outer labia in high BMI and average BMI subjects

Site	BMI status	mean	st. dev.	*p* value
exposed skin	average BMI	9.756	8.377	0.040
	high BMI	5.560	3.313	
outer labia	average BMI	19.893	14.995	0.175
	high BMI	25.667	17.543	

*p-*values reported in Table 2 were calculated using a Student’s t-test (95% confidence interval)

### Bacterial community modulations in the vulva were associated with BMI

**Fig. 1:**
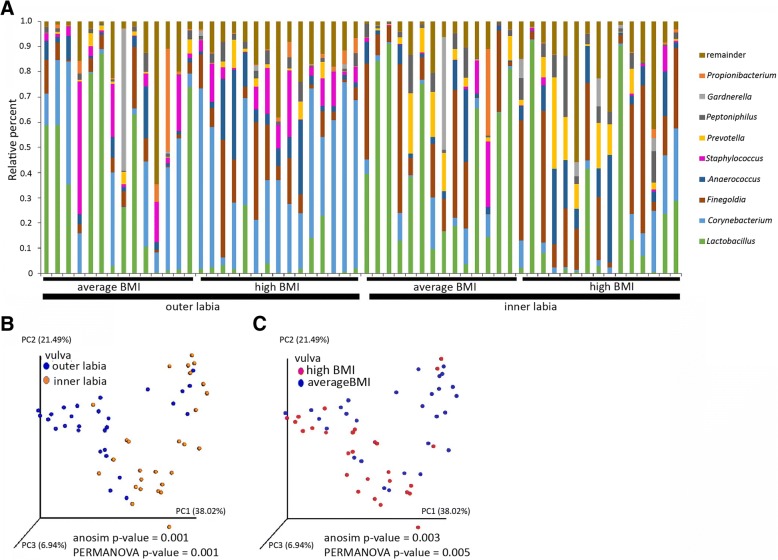
Microbiota of the inner and outer labia differ between average and high BMI women. The bar graph shows relative percent of bacterial genera for inner and outer labia (**a**). The top 13 most abundant genera (average across all labial samples) are shown. Weighted UniFrac principal coordinate analysis was done to show separation between microbiota of outer labia and inner labia (**b**), and average and high BMI women (**c**). Statistical significance of weighted UniFrac was calculated using anosim and PERMANOVA tests and *p*-values are shown

Given that pH can alter the growth of a variety of microorganisms [24–26], we next hypothesized that the resident microbiota of high BMI women would be altered compared to women of low BMI in the vulvar area due to the change in pH. This hypothesis was tested using 16S rRNA sequencing analysis. Figure 1a shows the relative abundance of top bacterial genera found in the vulva region (both the inner and outer labia). *Lactobacillus* was the most abundant genus, followed by *Corynebacterium*, across all vulva samples (both inner and outer labia); however, when calculated individually *Corynebacterium* was the most abundant species in the outer labia and *Lactobacillus* was the most abundant species in the inner labia. Beta diversity analysis, conducted using weighted UniFrac analysis, showed that while there was overlap, community populations of the outer labia (labia majus) clustered distinctly from the inner labia (labia minus) in agreement with previous reports [27–30] (Fig. 1b). Furthermore, *Finegoldia* and *Lactobacillus* were statistically more prevalent in the inner labia, while *Corynebacterium* and *Staphylococcus* were more prevalent in outer labia (Table 3). Beta diversity analysis using weighted UniFrac showed a distinct subpopulation of average BMI women that harbored bacterial communities that clustered separately from those of women with high BMI (Fig. 1c) while the majority average BMI women overlapped with the high BMI women. Statistical analysis of weighted UniFrac showed significant differences in vulvar bacterial communities between the inner and outer labia and average and high BMI women (Fig. 1b and c). Unweighted UniFrac analysis showed similar results (Additional file 1: Figure S1). Within the vulva (both inner and outer labia), women with average BMI had a statistically higher abundance of *Lactobacillus*, while *Corynebacterium* and *Anaerococcus* were more prevalent in high BMI women (Table 4). Within the inner and outer labia, women with average BMI had a statistically higher proportion of *Lactobacillus* (Tables 5 and 6), while *Corynebacterium* were more prevalent in high BMI women in the outer labia alone (Table 5). Shannon and Simpson’s diversity index calculations showed differences in vulvar communities with respect to average and high BMI (Table 7). Alpha diversity analysis of inner versus outer labia showed mixed results using the two indexes (Table 8). These data indicate that changes in the vulvar microbiome may be associated with BMI status.

**Table 3:** Relative abundance of top five most abundant genera within the vulva

Genus	Site	Mean	Standard Deviation	*p*-Value
*Corynebacterium*	outer labia	0.32	0.15	0.00
	inner labia	0.07	0.02	
*Finegoldia*	outer labia	0.10	0.11	0.02
	inner labia	0.22	0.19	
*Lactobacillus*	outer labia	0.20	0.28	0.05
	inner labia	0.32	0.33	
*Staphylococcus*	outer labia	0.09	0.11	0.00
	inner labia	0.02	0.06	
*Anaerococcus*	outer labia	0.07	0.09	0.65
	inner labia	0.09	0.10	

*p*-values reported in Table 3 were calculated using the Kruskal-Wallis Test

**Table 4:** Relative abundance of top five most abundant genera within the vulva

Genus	BMI	Mean	Standard Deviation	*p*-Value
*Corynebacterium*	average	0.13	0.16	0.04
	high	0.25	0.24	
*Finegoldia*	average	0.13	0.15	0.12
	high	0.19	0.17	
*Lactobacillus*	average	0.40	0.32	0.00
	high	0.13	0.24	
*Anaerococcus*	average	0.05	0.06	0.01
	high	0.11	0.11	
*Staphylococcus*	average	0.06	0.12	0.86
	high	0.05	0.07	

*p*-values reported in Table 4 were calculated using the Kruskal-Wallis Test

**Table 5:** Relative abundance of selected genera within the outer labia

Genus	BMI	Mean	Standard Deviation	*p*-value
*Corynebacterium*	average	0.20	0.19	0.01
	high	0.42	0.21	
*Lactobacillus*	average	0.36	0.33	0.03
	high	0.05	0.09	

*p*-values reported in Table 5 were calculated using the Kruskal-Wallis Test

**Table 6:** Relative abundance of selected genera within the inner labia

Genus	BMI	Mean	Standard Deviation	*p*-value
*Corynebacterium*	average	0.05	0.06	0.49
	high	0.08	0.10	
*Lactobacillus*	average	0.44	0.32	0.02
	high	0.21	0.31	

*p*-values reported in Table 6 were calculated using the Kruskal-Wallis Test

**Table 7:** Alpha diversity of vulvar bacterial communities by BMI

Alpha diversity	BMI	Mean	Standard Deviation	*p*-value
Shannon index	average	3.57467	0.95033	0.00010
	high	4.56874	0.91445	
Simpson’s index	average	0.78096	0.15236	0.00003
	high	0.89420	0.90357	

*p*-values reported in Table 7 were calculated using the Kruskal-Wallis Test

**Table 8:** Alpha diversity of vulvar bacterial communities by site

Alpha diversity	Site	Mean	Standard Deviation	*p*-value
Shannon index	outer labia	4.39629	0.89511	0.01052
	inner labia	3.78141	1.11852	
Simpson’s index	outer labia	0.86943	0.10387	0.13750
	inner labia	0.80964	0.18789	

*p*-values reported in Table 8 were calculated using the Kruskal-Wallis Test

### Predicted metabolomic profiles of microbiota from high and average BMI women

**Fig. 2:**
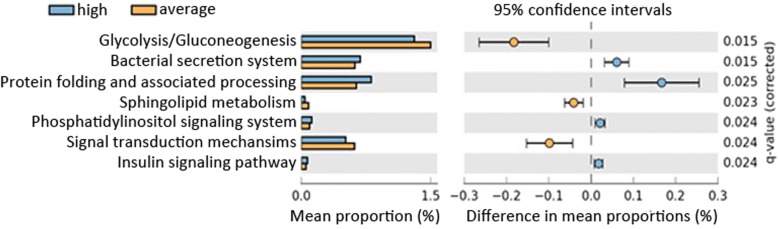
Predicted metabolic differences associated with high and average BMI in microbial communities found in the vulva. Metabolic pathways were identified using PICRUSt software. Welch’s t-test with a Benjamini-Hochberg FDR test correction was used to calculate q-value

In order to examine whether high and low BMI state affected the metabolic activity of microbial communities in the vulva, we utilized the PICRUSt algorithm [31] to infer their metagenomes and collapse the genes into Kyoto Encyclopedia of Genes and Genomes (KEGG) pathways at level 3 (Fig. 2). Figure 2 shows that the glycolysis/gluconeogenesis pathway was predicted to be enriched in average BMI women, while bacterial secretion systems were predicted to be enriched in high BMI women.

### Abdominal skin microbiota was not affected by BMI status

**Fig. 3:**
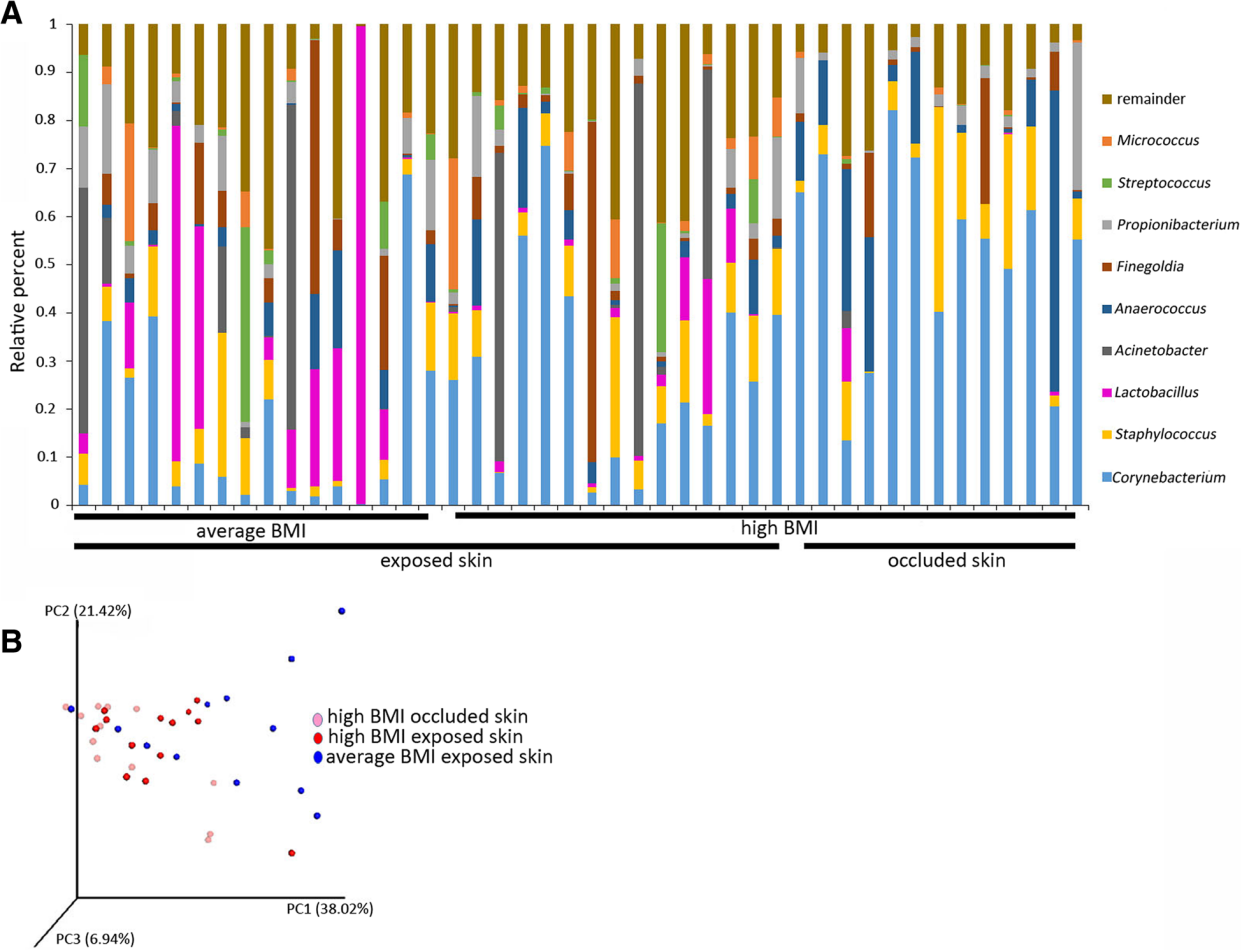
Microbiota of occluded and exposed abdominal skin were similar and not affected by BMI status. The bar graph (**a**) shows relative percent of bacterial genera for each sample collected from the abdomen. The top ten most abundant genera (average across all abdominal samples) are shown. Weighted UniFrac principal coordinate analysis shows microbiota of occluded and exposed skin were similar and not modulated by BMI status (**b**)

In order to understand whether the BMI-associated microbiome shift was localized to the vulva or extended to an adjacent region, 16S rRNA analysis was performed on microbiota samples from exposed and occluded abdominal skin. Samples from exposed skin were taken from the lower abdomens of women with average and high BMI. Occluded skin samples were taken from skin folds of women with high BMI. Figure 3a shows the percent abundance of the top nine most abundant genera for exposed and occluded skin. There was a high abundance of *Staphylococcus* and *Corynebacterium* across all samples, which is consistent with previous reports characterizing skin microflora [13–16, 32]. Despite an observed change in TEWL values (Table 2), the relative abundance of *Corynebacterium*, *Lactobacillus*, and *Micrococcus* in exposed skin were not statistically different between the normal and high BMI groups (Table 9). This was not surprising because TEWL values of both groups were well within the healthy range [22, 23].

**Table 9:** Relative abundance of top three most abundant genera of exposed abdominal skin

genus	BMI	Mean	Standard Deviation	*p*-value
*Lactobacillus*	average	0.19	0.29	0.25
	high	0.04	0.08	
*Corynebacterium*	average	0.16	0.19	0.06
	high	0.28	0.20	
*Staphylococcus*	average	0.07	0.08	0.97
	high	0.10	0.07	

*p*-values reported in Table 9 were calculated using the Kruskal-Wallis Test

In a similar study of abdominal skin microflora, Edwards et al. found a high abundance of *Paenibacillus*, an endospore-forming bacteria typically found in soil, on exposed and occluded abdominal skin [33] that was not observed in this study. Edwards et al. sampled only women with a BMI ≥40, while the current study sampled women with a BMI of 18–25 or ≥ 30. This difference in subject pool could account for the failure to detect *Paenibacillus*.

Principle coordinate analysis using weighted UniFrac scores showed significant overlap between exposed and occluded skin for both normal and high BMI women (Fig. 3b). These data indicate that microbiomes of non-mucosal skin, like the abdomen, may be more stable and resistant to BMI-induced physiological changes.

## Discussion

Increased BMI has been associated with hypertension, coronary heart disease, type-2 diabetes, stroke, gall bladder disease, osteoarthritis, sleep apnea, metabolic syndrome, and poor quality of life [34, 35]. Additionally, it has been shown that obesity may impact the gut microbiome causing a change in diversity and metabolic pathways [7–10]. Metabolic syndrome, which is associated with high BMI, has been shown to have a profound influence on insulin resistance and associated carbohydrate metabolism and genesis. Specifically, it has been shown that those conditions reduce glycogen levels while increasing plasma glucose levels [36, 37]. It is hypothesized that these altered conditions can have a profound effect on the structure of the vulvar microbiome.

Microbiome changes can result from both environmental and host-associated factors. With regard to environmental factors, it was observed that vulvar pH was different between normal and high BMI subjects. Conversely, BMI did not affect the health of the skin barrier, which would imply similar water of activity for all subjects. These data indicate that BMI alters specific biophysical properties of the labial skin like pH; however, BMI does not impact non-occluded skin of the abdomen, indicating that the effect is not a global phenomenon but may be specific to mucosal sites.

Within the vulva (both inner and outer labia), women with normal BMI had a statistically higher abundance of *Lactobacillus*, while *Corynebacterium* and *Anaerococcus* were more prevalent in high BMI women (Table 4). Women with average BMI had a statistically higher proportion of *Lactobacillus* in the inner and outer labia (Tables 5 and 6), while *Corynebacterium* were more prevalent in high BMI women in the outer labia alone (Table 5). These data indicate that changes in the vulvar microbiome may be associated with BMI status. Colonization of the vaginal tract, directly adjacent to the inner labia, is well documented and this area is known to harbor a variety of bacteria with *Lactobacillus* species often predominating in healthy women [38–41]. This suggests that the vulva may be a transitional zone whereby bacterial communities differ from interior to exterior, with the interior resembling vaginal microbiota and the exterior resembling skin microbiota. It is important to note that while differences in the vulvar microbiome were correlated to BMI, there are other potential factors that could influence the microbiome state that were not controlled for in this study like ethnicity and diet. More studies are needed to determine how these factors could alter the vulvar microbiome.

One explanation for the microbiome shift in high BMI subjects could be alterations of the carbon balance provided to the community by the host. *Lactobacillus* is well known for its ability to alter the pH of its environment. Provided the right conditions and appropriate carbohydrates for fermentation, *Lactobacillus* will produce lactic acid that will acidify the environment [42]. This process provides a competitive advantage to *Lactobacillus* with respect to their ability to thrive in environments where other bacteria, such as *Escherichia coli*, cannot [43–45]. However, these conditions appear to have changed with regard to the high BMI subjects.

The PICRUSt algorithm was employed to investigate metabolic function based on inferred metagenomes. It is important to note that while the PICRUSt algorithm is a validated method to infer metagenomes from genus level 16S rRNA gene analysis it does not replace the accuracy of deep metagenomic analysis which could detect the metabolomic variation that can happen at the species and strain level. Upon analysis of the communities’ metabolic patterns using PICRUSt, decreased glycolysis/gluconeogenesis was observed among high BMI subjects (Fig. 2), which implies a change in the carbon source available to the consortia. Metabolic syndrome is known to increase serum glucose and decrease glycogen levels. A previous report demonstrated a positive correlation between *Lactobacillus* abundance and glycogen levels in the vagina [46]. Thus, reduced glycogen levels would put *Lactobacillus* at a disadvantage compared with other members of the community. Another potential factor that may have impacted *Lactobacillus* abundance would be the hosts’ expression and activity of alpha-amylases or other enzymes required to cleave glycogen. Lactobacillus requires host enzymes to break down glycogen into subunits that it can readily utilize [47], therefore, a decrease in alpha-amylase expression or activity in high BMI women would put *Lactobacillus* at a disadvantage. More studies are needed to determine if amylase activity or expression is altered in high BMI women.

PICRUSt (Fig. 2) results imply that other community members are more metabolically active with respect to glucose utilization as a result of the host response to increased BMI. This study did not quantitatively measure the metabolic activities of individual members in the community so further experimentation would be needed to confirm this hypothesis. The increase in insulin signaling pathway genes (Fig. 2) in high BMI subjects further strengthens the association between an underlying metabolic syndrome in those subjects and impacts on the microbiome. Metabolic syndrome was not clinically assessed for these subjects so future studies are needed.

## Conclusion

The BMI-induced changes observed in this study provide insight regarding regulation of microbiomes through host-community interactions. The loss of appropriate carbon sources appears to have limited *Lactobacillus* proliferation. The presumed loss of glycogen and resulting loss of pressure exerted by *Lactobacillus* allowed less predominant microbes to proliferate. A concomitant increase in glucose allowed dominance by other members of the community. The result was an observed increase in skin pH and decrease in *Lactobacillus* predominance associated with high BMI. Further studies are warranted, including direct measurement of glycogen/glucose levels and collection of additional subject health data to assess the presence of metabolic syndrome. It may also be prudent to include microbiome and associated metabolic assessments as an additional means to predict metabolic syndrome in humans. Furthermore, data suggest that adding carbon/nitrogen sources that are directly utilizable by *Lactobacillus* and not by other members of the community may help to sustain the vulvar microbiome in a more *Lactobacillus*-dominant state.

## Material and methods

### Sample collection

For details on how samples were collected see Additional file 2. In short, 40 generally-healthy female subjects aged 18–35 years were enrolled, including 20 subjects (50%) with normal BMI (18–25) and 20 subjects (50%) high BMI (≥30). Subjects self-reported having regular menstrual cycles for the preceding three months, had no history of skin disorders or abnormal skin conditions in the anatomical region of interest, and had no open cuts or body art in the lower torso (waist to knees). Full inclusion and exclusion criteria for this study can be found in Additional file 2. Patients volunteered to participate in the study and signed a written informed consent. Subjects were pre-screened using a script approved by the Institutional Review Board. During the first visit, subjects completed the informed consent, were screened for inclusion and exclusion criteria, and their BMI was measured. If inclusion criteria were met, the subject was asked to return between days 14 and 21 of the menstrual cycle for biophysical measurement and collection of microbiota swabs. During visit 2, TEWL and pH were measured on exposed abdominal skin, inside the abdominal skin fold (high BMI women only) and the labium majus. Swabs were collected at non-overlapping sites on exposed abdominal skin, inside the abdominal skin fold (high BMI women only), and from the labium majus and labium minus. DNA was extracted and microbiota composition was determined by 16S rRNA barcode modified Tag-encoded FLX amplicon pyrosequencing (bTEFAP™) using a previously described protocol [48]. Sequences were submitted to the Sequence Read Archive (SRA) under accession number SRP116641.

### Data analysis

The Quantitative Insights into Microbial Ecology (QIIME) 1.9.0 data analysis tool was used for used for subsequent 16S rRNA data analysis. Sequences were de-mutiplexed and operational taxonomic unit (out) tables constructed using closed reference OTU picking against the Greengenes reference database (version 13_8) at 97% identity, and the remaining OTUs were discarded [49]. After quality filters there were a total of 865,634 reads. An average of 7154 counts per sample. Samples with less than 1020 counts were excluded from the analysis. Rarefaction curves showed that very few additional OTUs were observed after 1020 reads. Taxonomy was assigned based on the Greengenes reference database. Alpha diversity metrics were calculated in QIIME 1.9.0. Beta diversity evaluations were done in QIIME 1.9.0 using weighted and unweighted UniFrac metrics and visualized using principle coordinates analysis (PCoA) [50, 51]. PICRUSt was used to predict metagenomes and collapse the genes into KEGG pathways (level 3).

### Statistical analysis

Linear regression and t-tests were used to determine whether pH and TEWL were related to BMI status. Significance was defined at *p* ≤ 0.0500. Statistical Analysis of Metagenomic Profiles (STAMP) was used to determine differences in the KEGG functions between the high and average BMI groups. Welch’s t-test with a Benjamini-Hochberg FDR test correction was used to calculate q-value. Differences in alpha diversity metrics and relative abundance of bacterial genera were calculated using Kruskal-Wallis tests. Differences in beta diversity metrics were calculated using anosim and PERMANOVA tests. *P* values less than or equal to 0.05 were considered significant and values were reported.

## Additional files

Additional file 1: **Figure S1.** Unweighted UniFrac principal coordinate analysis was done to show separation between microbiota of outer labia and inner labia (a), average and high BMI of the outer labia (B) and inner labia (C). (JPG 97 kb)

Additional file 2: Study Protocol. (PDF 337 kb)

## Abbreviations

BMI: Body mass index
IRB: Institutional review board
KEGG: Kyoto encyclopedia of genes and genomes
OTU: Operational taxonomic units
PCoA: Principal coordinates analysis
PCR: Polymerase chain reaction
PICRUSt: Phylogenetic investigation of communities by reconstruction of unobserved states
QIIME: Quantitative Insights into microbial ecology
rRNA: Ribosomal ribonucleic acid
SRA: Sequence read archive
TEWL: Trans-epithelial water loss
