# Supplementary figures and images for: Comparative study of vulva and abdominal skin microbiota of healthy females with high and average BMI
Source: BMC Microbiol. 2019 Jan 17;19:16. doi: 10.1186/s12866-019-1391-0 (PMC6337831; doi:10.1186/s12866-019-1391-0)

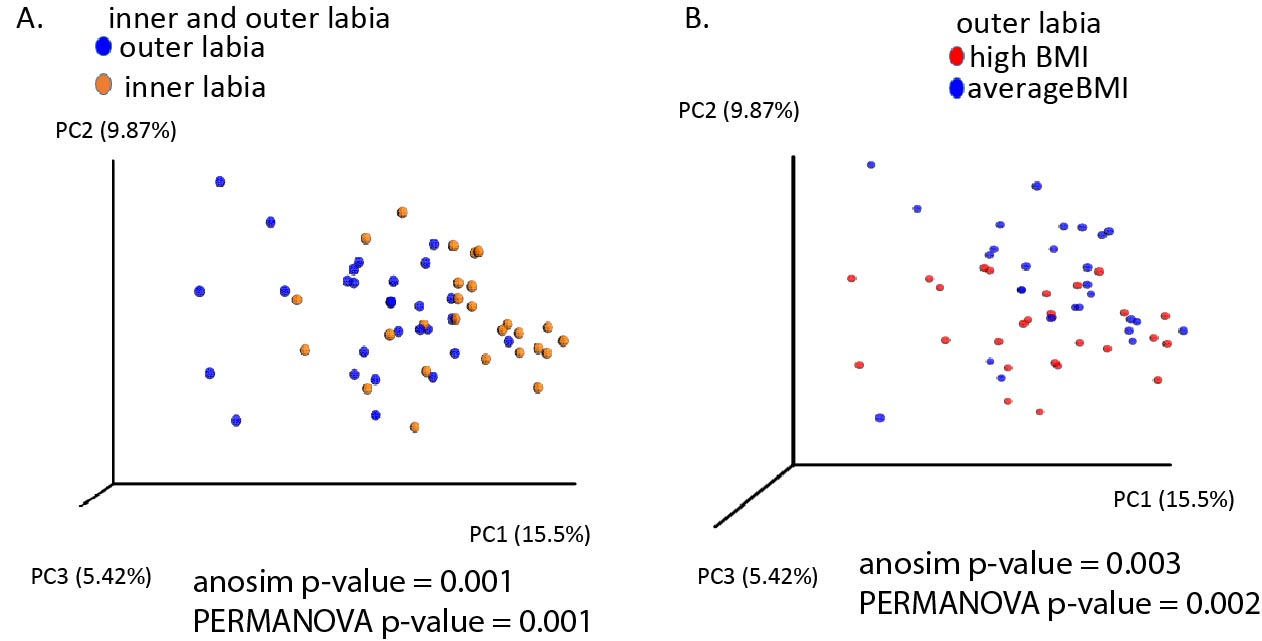

Supplement: Supplementary file 1 — Figure S1. Unweighted UniFrac principal coordinate analysis was done to show separation between microbiota of outer labia and inner labia (a), average and high BMI of the outer labia (B) and inner labia (C). (JPG 97 kb) [file 12866_2019_1391_MOESM1_ESM.jpg]
