# Supplementary material for: Comparative study of vulva and abdominal skin microbiota of healthy females with high and average BMI
Source: BMC Microbiol. 2019 Jan 17;19:16. doi: 10.1186/s12866-019-1391-0 (PMC6337831; doi:10.1186/s12866-019-1391-0)
Supplement: Supplementary file 2 — Study Protocol. (PDF 337 kb) [file 12866_2019_1391_MOESM2_ESM.pdf]

Assessment of Skin Micro Ecology in Females

Testing Equipment

All equipment used in this study is safe for use on human skin. Manufacturer instructions for use will be followed. The use of all equipment listed below will follow the manufacturer's recommended operational procedures. Equipment will be monitored during the study to ensure reliability of data and operational characteristics. Manufacturer recommendations for equipment/probe sanitizing between subjects will be followed.

- Epidermal Water loss will be measured with the Dermalab® Transepidermal Water Loss Module (Cortex Technology, Denmark). Disposable, individual probe covers will be used for collection of EWL/TEWL measurements.
- Skin pH will be determined with the Courage and Khazaka pH meter (Cologne, Germany). The pH probe will be immersed in a 10% bleach solution for a minimum of 10 minutes between each subject and rinsed thoroughly with deionized water and stored in deionized water when not in use.
- pH calibration is completed prior to study start. Each day of the study the calibration is verified in triplicate with pH 4 and pH 7 standard solutions. If the verification data is out of range (± 0.1 pH), the probe will be recalibrated. All calibration and verification documentation will be filed in the study binder.

EXPERIMENTAL DESIGN

This study is a single center, non-randomized study.

Each subject will make two (2) visits to the testing site to complete the study.

- Visit 1 – Consenting and Screening (approximately 1 hour visit)
- Visit 2 –Biophysical measurements (approximately 2.5 hour visit)

The testing study visit (Visit 2) will be completed when the subject is between menstrual cycle days 14 and 21.

STUDY POPULATION

A sufficient number of female subjects who meet the following inclusion/exclusion criteria will be recruited for this study to allow for completion of approximately 20 subjects with normal BMI (18-25) and 20 subjects with high BMI (≥30). Subjects will be pre-screened by an outside recruiting agency, according to the pre-screening script approved by the Institutional Review Board.

INCLUSTION CRITERIA

Potential subjects must satisfy the following criteria before participating in the study:

1. Fluent in English and have read, understood, and signed the ICF and HIPPA form;
2. General good health by self-report;
3. Female, 18-35 years of age;
4. Have regular menstrual cycles of 26-35 days for the past 3 months;

5. Between menstrual cycle day 14 and 21 for sample collection visit (Visit 2);
6. Have a BMI of 18-25 or ≥30.00 (per NIH online BMI calculator[1]);
7. Have intact skin in the perineal region, as self-reported at the time of consenting;

EXCLUSION CRITERIA

Potential subjects who meet any of the following self-reported criteria will be excluded from participating in the study:

1. Be pregnant, trying to become pregnant or lactating ;
2. Have history of or current treatment for a skin disorder/abnormal skin condition of the anatomical regions to be tested that may confound the results of the study such as:
    a. Eczema
    b. Psoriasis
    c. Dermatitis
    d. Acne
    e. Any other form of dermatologic conditions
3. Have open or healing cuts/incisions, abrasions or broken skin in the abdominal area in the last 6 weeks;
4. Have body piercings or tattoos in the lower torso area (waist to knees);
5. Be using or have used antibiotics, antifungals, steroids or antivirals (oral or topical in the testing areas) less than 6 weeks prior to Visit 2;
6. Have a history of or current, self-reported genital herpes;
7. Have current or in the past 6 weeks, conditions such as:
    a. Active vaginal infection
    b. Urinary tract infection
    c. Unusual or foul-smelling vaginal discharge
    d. Urethral discharge
    e. Vaginal pain
    f. Urogenital pain
    g. Pelvic pain
8. Have used lotion, powder, ointment, cream, perfume or oil on the skin in the testing areas region 48 hours prior to Visit 2;
9. Be using douching substances, vaginal medications, suppositories, and feminine deodorant spray, wipes, or lubricants to the genital area 48 hours prior to Visit 2;
10. Sexual intercourse less than 48 hours prior to Visit 2;
11. Current treatment for any skin conditions on the testing area;
12. Have bathed, showered, exercised or have gone swimming within 4 hours of Visit 2;
13. Have shaved, waxed or used depilatory treatments in the abdominal and/or perineal area in the last 72 hours.
14. Have smoked or consumed anything containing nicotine or caffeine less than 2 hours prior to Visit 2;
15. Have Type I or Type II diabetes;
16. Have hepatitis or be HIV positive;
17. Have other medical conditions (such as an immunosuppressive condition) which in the Investigator's opinion would compromise their participation in the study;
18. Current enrollment in another clinical study.

---

[1] http://www.nhlbisupport.com/bmi/

RANDOMIZATION

The study will not be randomized.

STUDY PROCEDURES

Visit 1

1. Subjects who report to the testing center will be given a copy of the Informed Consent and ample time to read the information. Study personnel will review the details of the study with each subject. After all questions have been answered and the subject has agreed to participate, she will sign the Informed Consent and HIPPA form. The original signed consent shall be retained by the site in the study file. A copy of the signed consent form shall be given to the subject.
2. Subjects will be screened according to the inclusion/exclusion criteria.
3. The subject's weight and height will be obtained, recorded and the BMI calculated using the National Institute of Health online BMI calculator
    a. Height will be measured to the nearest ½ inch
    b. Weight will be measured to the nearest ½ pound
4. Subjects who meet all the inclusion and none of the exclusion criteria will be shown the instruments and supplies to be used to collect measurements and samples during the study.
5. Subjects will be asked to provide a list of all over the counter and prescription medications they have taken in the past 6 weeks.
6. Study coordinator will schedule Visit 2 to occur when the subject is between menstrual cycle days 14 and 21.
7. Subjects will be given an instruction sheet on personal products and activities to avoid prior to Visit 2.

Visit 2

1. The subject will return to the testing site and escorted to a private area where they will be asked to undress from the waist down. She will lie on an exam table with her feet in the stirrups covered with a privacy drape.
2. A visual inspection of the testing areas for exclusionary skin disorders that would prohibit further testing will be assessed and noted.
3. Subjects will be enrolled if no exclusionary criteria are noted.
4. After at least a 15 minute acclimation period, the subject's hips will be elevated using the exam table lift and a pillow may be placed under the hips (modified Trendelenberg position) to allow for the TEWL probe placement at a horizontal angle no greater than 45 degrees during instrumental testing procedures. This is to ensure that full contact is made with the skin and to allow for passive diffusion of water vapor through the evaporimeter chamber.
5. Sites may be marked with a skin marking pen to prevent overlap of swab sampling areas with instrument placement.
6. The evaporimeter probe will be placed on each site in the following order (See Figure 1 and 2):
    a. Site A: Exposed abdominal skin – approximately 4cm by 2 cm area 2-3 inches below the umbilicus)
    b. Site B: Inside abdominal skin fold – approximately 4cm by 2cm area;
        i. Note: Site B only to be tested on high BMI subjects
    c. Site E: Right labium majus lateral to the introitus

7. A 1-minute TEWL measurement will be taken with 30 seconds stabilization and 30 seconds data collection for each site. A plastic probe cover will be replaced after each reading for the site.
8. The pH probe will be placed on each site but will not overlap TEWL testing site in the following order (See Figure 1 and 2)
    a. Site A – Exposed abdominal skin – approximately 4cm by 2 cm area 2-3 inches below the umbilicus)
    b. Site B: Inside abdominal skin fold – approximately 4cm by 2cm area;
        i. only be sampled/tested on high BMI subjects
    c. Site F: Left labium majus lateral to the introitus (Figure 2).
9. Three pH measurements will be taken consecutively. The probe will be in contact with the skin briefly (about 3 seconds for each reading).

10. The pH probe will be immersed in a 10% bleach solution between subject usage and rinsed thoroughly with deionized water and stored in deionized water when not in use.

11. The following anatomical areas will be swabbed for skin microbial ecology and sample areas will not overlap the TEWL/pH sites:
    a. Site A: Exposed abdominal skin – approximately 4cm by 2 cm area 2-3 inches below the umbilicus)
        i. Note: avoid scar tissue and skin folds if present.

    b. Site B: Inside abdominal skin fold – approximately 4cm by 2cm area;
        i. Note: Site B sampled for high BMI women only

    c. Site C: Left and right labium majus near the introitus approximately from a 2cm by 2cm area (one swab sampled on the left and a second swab sampled on right to be combined in the same test tube)

    d. Site D: Left and right labium minus near the introitus – ~2cm by 2cm area (one swab sampled on the left and a second swab sampled on right to be combined in the same test tube)

12. The two swab sampling procedure to be followed for all four sites:
    a. Dip a sterile swab (FLOQSwabs by Copan) into 9 ml of a pre-reduced dilution blank. The swab is rolled for 30 seconds (two complete turns) and placed into a sterile micro-centrifuge tube where the handle is broken off. The sample will not be taken from the TEWL/pH site.
    b. A second swab is dipped into the *same* 9 ml dilution blank and the contralateral skin region is then sampled by rolling the swab for 30 seconds (two complete turns). The second swab is also broken off into the original micro-centrifuge tube.
    c. Discard the used dilution blank
13. There will be a total of 3 swab sites for normal BMI and 4 swab sites for high BMI subjects
14. Samples will be placed in Clinical Research Services (CRS) -20° C lab freezer within 15 minutes of collection.
15. The subject will redress and be dismissed from the study as this concludes their participation in the study.
16. Samples to be shipped by the sponsor to an outside lab for analysis.
    a. Shipping Procedure (to be completed by the sponsor)
        i. Place the micro-centrifuge tube is placed in a transport bag labeled with subject number, site of sample, and date / time collected.

ii. Hold samples at a minimum of -20° C until time of shipment. Samples to be shipped overnight with shipping dates to be determined by the sponsor.

17. The analysis of the swab samples is expected to take approximately 10-12 weeks. Results from the DNA analysis will be reported to the sponsor and site. The Medical Investigator will review results from the analysis. If in the opinion of the Medical Investigator the results indicate a condition that needs follow up, the subject will be contacted by the Medical Investigator to discuss those findings with the subject. The subject will be provided the option to be referred to her healthcare provider for follow up medical care.

**Procedure**	**Visit 1**	**Visit 2 – Abdominal/Vulvar Sampling**
Informed Consent	X	
Inclusion/Exclusion	X	
Medical History/Concomitant Medications	X	X
Adverse Event(s)		X
pH testing		X
TEWL		X
Swabs for microbial DNA analysis		X
Medical follow-up discuss (if necessary)		After results are reported

Note: Visits 1 and 2 may be conducted on the same date.
